# Can Intelligence Affect Alcohol-, Smoking-, and Physical Activity-Related Behaviors? A Mendelian Randomization Study

**DOI:** 10.3390/jintelligence11020029

**Published:** 2023-01-31

**Authors:** Hansen Li, Xing Zhang, Xinyue Zhang, Zhenhuan Wang, Siyuan Feng, Guodong Zhang

**Affiliations:** 1 Key Laboratory of Physical Fitness Evaluation and Motor Function Monitoring, Institute of Sports Science, College of Physical Education, Southwest University, Chongqing 400715, China; 2 Graduate School, University of Wisconsin-Madison, Madison, WI 53706, USA; 3 Institute for Health and Sport (iHeS), Victoria University, Melbourne, VIC 3011, Australia; 4 Laboratory of Genetics, University of Wisconsin-Madison, Madison, WI 53706, USA

**Keywords:** problematic behavior, health, exercise, lifestyle, abuse

## Abstract

People with high levels of intelligence are more aware of risk factors, therefore choosing a healthier lifestyle. This assumption seems reasonable, but is it true? Previous studies appear to agree and disagree. To cope with the uncertainty, we designed a mendelian randomization (MR) study to examine the causal effects of genetically proxied intelligence on alcohol-, smoking-, and physical activity (PA)-related behaviors. We obtained genome-wide association study (GWAS) datasets concerning these variables from separate studies or biobanks and used inverse-variance weighted (IVW) or MR-Egger estimator to evaluate the causal effects according to an MR protocol. The MR-Egger intercept test, MR-PRESSO, and funnel plots were employed for horizontal pleiotropy diagnosis. The Steiger test (with reliability test), Cochran’s Q test, MR-PRESSO, and leave-one-out method were employed for sensitivity analysis. We found significant or potential effects of intelligence on alcohol dependence (OR = 0.749, *p* = 0.003), mental and behavioral disorders due to alcohol (OR = 0.814, *p* = 0.009), smoking (OR = 0.585, *p* = 0.005), and smoking cessation (OR = 1.334, *p* = 0.001). Meanwhile, we found significant or potential effects on walking duration (B = −0.066, *p* < 0.001), walking frequency (B = −0.055, *p* = 0.031), moderate PA frequency (B = −0.131, *p* < 0.001), and vigorous PA frequency (B = −0.070, *p* = 0.001), but all in a negative direction. In conclusion, our findings reinforce some existing knowledge, indicate the complexity of the health impacts of human intelligence, and underline the value of smoking and alcohol prevention in less intelligent populations. Given the existing limitations in this study, particularly the potential reverse causality in some estimations, re-examinations are warranted in future research.

## 1. Introduction

Intelligence is a complex trait that contains multiple dimensions (Colom et al. 2010). According to a widely-accepted definition, intelligence can be described as “a very general mental capability that, among other things, involves the ability to reason, plan, solve problems, think abstractly, comprehend complex ideas, learn quickly and learn from experience. It is not merely book learning, a narrow academic skill, or test-taking smarts. Rather, it reflects a broader and deeper capability for comprehending our surroundings—‘catching on’, ‘making sense’ of things, or ‘figuring out’ what to do” (Gottfredson 1997). In the past decades, a number of strategies have been developed to measure intelligence (Deary 2001; Deary et al. 2010), including mental tests and some emerging imaging methods such as fMRI (Colom and Thompson 2011; Saxe et al. 2018; Tong et al. 2022). Numerous studies and their following replications have collectively suggested the effectiveness of intelligence measurement, which enables subsequent studies on the associations between intelligence and various health outcomes.

Owing to the genetic foundation (Davies et al. 2011), intelligence is believed to stay stable in rank order across the lifespan (Deary et al. 2006, 2010). Meanwhile, individuals vary widely in intelligence (Deary et al. 2009, 2010). These may explain variations in the incidence of some long-term events, for example, mortality (Calvin et al. 2011; Deary et al. 2019). To reveal the mechanisms behind the phenomenon, scholars are trying to find out potential mediators on this pathway. For example, a better socioeconomic status associated with higher intelligence may reduce mortality (Arden et al. 2016; Davies et al. 2011). Furthermore, some more downstream health outcomes, such as cardiovascular diseases and cancers (Backhouse et al. 2017; Calvin et al. 2017), may also link intelligence and mortality. Notably, behavioral factors are believed to play essential roles. Deary et al. (2019) suggest that higher intelligence is related to a higher likelihood of engaging in healthier behaviors, such as not smoking, quitting smoking, not binge drinking, and exercising more. These behavioral factors may effectively reduce mortality and contribute to understanding the long pathway.

To date, some observational studies with one measurement point have documented the associations between intelligence and health-related behaviors such as alcohol use and smoking, but the reported associations were not all in a beneficial direction (Mortensen et al. 2005; Salber et al. 1962; Sjölund et al. 2012; Suhaimi et al. 2019; Wraw et al. 2018). These uncertainties underline the necessity of further studies. Since these results were derived from observational studies, excluding the likelihood of reverse causality and confounding is nearly impossible (Sekula et al. 2016; Zhu et al. 2018), particularly given the possible effect of education on intelligence (Davies et al. 2019; Ritchie and Tucker-Drob 2018). By comparison, Mendelian randomization (MR) can be a new option for this research topic. A common principle of Mendelian randomization is that if a genetic variant (gene) influences an environmentally modifiable risk factor that itself alters disease risk, then the genetic variant should be associated with disease risk, and the causal effect of the environmentally modifiable risk factor on disease risk can be calculated under certain requirements (Smith et al. 2007). In practice, Mendelian randomization uses genetic variants as instrumental variables to evaluate the causal effects of modifiable exposures (risk factors) on outcomes (e.g., health, social, and economic variables) (Richmond and Smith 2022). In other words, Mendelian randomization offers a special mediation model in which genetic variants are linked to the outcome only through the exposure. On the premise of some assumptions for excluding other pathways, we can use the genetic variant-outcome and the genetic variant-exposure associations to evaluate the exposure-outcome association. As Mendel’s Laws of Inheritance dictate, alleles segregate randomly from parents to offspring, so the potential confounding of the exposure–outcome association can be ruled out (Richmond and Smith 2022; Zheng et al. 2017). This advantage makes Mendelian randomization somewhat similar to randomized controlled trials (Bennett and Holmes 2017). Therefore, many recent studies have used the MR method to investigate the impact of intelligence on health outcomes (Davies et al. 2019; Li et al. 2021). For the same reason, we designed this MR study to examine the causal effects of genetically proxied intelligence on alcohol-, smoking-, and physical activity-related behaviors and, in turn, offer insights into the health impacts of human intelligence.

## 2. Materials and Methods

### 2.1. Design and Population

We employed a two-sample Mendelian randomization (2SMR) design (Hemani et al. 2018), which is a typical MR method that obtains the genetic variant-exposure and genetic variant-outcome associations from two samples of participants (Lawlor 2016). To control population stratification bias, we selected individuals of European ancestry only for the current analyses (Lin et al. 2022). Since this study was based on publicly available summary-level data, informed consent and ethical approvals were not applicable.

### 2.2. Variables and Data Sources

Our datasets were from biobanks or individual studies. Our exposure dataset concerning intelligence was a meta-analysis of 14 cohorts (Savage et al. 2018). The intelligence phenotype was measured by multiple tests, for example, fluid-intelligence test (assessing the ability to solve problems by abstraction) (Chen et al. 2019), Stroop (assessing cognitive abilities) (Criscuolo et al. 2019; Horička et al. 2020), and SAT test (a scholastic assessment test related to general cognitive ability) (Frey and Detterman 2004).

Four alcohol-related datasets were obtained from the FinnGen biobank. The FinnGen biobank is a project that collects biological samples from 500,000 participants in Finland over six years with the aim of improving health through genetic research. The official descriptions of the variables (according to the FinnGen) are shown in Table 1, and the detailed descriptions can be found at https://r7.finngen.fi/ (accessed on 6 November 2022). These variables are coded in binary to indicate the incidence of these events.

Two smoking datasets were obtained from the FinnGen and the other three were obtained from a study offered by The Tobacco and Genetics Consortium (2010) (Table 1), all binary-coded.

Four physical activity (PA) datasets were obtained from the UK-biobank. Walking frequency refers to the number of days with walking (≥10 min) in a typical week. Walking duration refers to the time spent on walking within a typical day. The frequency of moderate or vigorous PA refers to the number of days with moderate or vigorous PA (≥10 min) in a typical week. These PA items were all captured by numeric text boxes and were coded as continuous variables.

### 2.3. Instrumental Variables (IVs) Selection

Genetic variants (SNPs) are used as instrumental variables in MR. We used a genome-wide significance threshold (*p* < 5 × 10^−8^) to select genetic instruments strongly associated with exposures (intelligence) (Burgess et al. 2019). Moreover, all initially identified genetic variants were clumped using PLINK to ensure that our instruments were from an independent set of variants (settings: clump-r^2^ = 0.001 and clump-kb = 10,000) (Purcell et al. 2007). We also screened the datasets and ensured that no SNP-outcome association reached a significance threshold. F-statistic for each instrument was estimated by F = beta^2^/SE^2^ (Chen et al. 2021; Feng et al. 2022; Rosa et al. 2019). IVs with an F-statistic < 10 were regarded as weak instruments (Burgess et al. 2015) and were excluded from the latter MR analysis.

### 2.4. Mendelian Randomization Analysis

**Figure 1:**
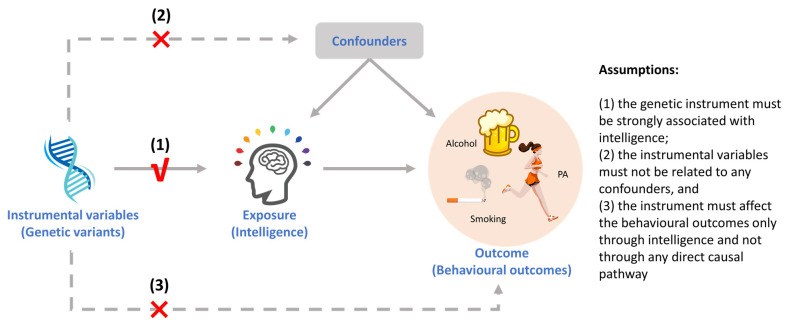
Schematic diagram of the MR assumption for this study.

There are three basic assumptions for running MR: (1) genetic variants should be strongly associated with the exposure; (2) genetic variants used as instruments must not be related to confounders; and (3) the genetic variants affect the outcome via the exposure only and not through any direct causal pathway (Davies et al. 2018; Yuan et al. 2020). A schematic diagram for this study is shown below (Figure 1).

The first assumption is easy to be validated by finding SNPs robustly associated with exposure. By comparison, the validation of the second and third assumptions is hard and even impossible (Chen et al. 2022a; Teumer 2018). To alleviate this issue, we removed any SNPs with potential pleiotropic effects through literature review and database searching (https://snipa.helmholtz-muenchen.de/snipa3/, accessed on 6 November 2022) (Chen et al. 2022b). 

Due to the difficulties in meeting these MR assumptions, assessing and addressing the threats to MR estimation is highly important. Theoretically, horizontal pleiotropy is the greatest threat that occurs when the variant has an effect on other traits outside of the pathway of the exposure and has an impact on the target outcome, or when the variant has a direct effect on the target outcome (Morrison et al. 2020; Verbanck et al. 2018). 

We used three methods to assess horizontal pleiotropy. The first method is the MR-Egger intercept test. The intercept of MR-Egger regression can be interpreted as the average horizontal pleiotropy of all IVs under the InSIDE assumption (assuming the independence between instrument strength and direct effects) (Burgess and Thompson 2017). An intercept not substantially different from zero can be evidence of no horizontal pleiotropy or balanced horizontal pleiotropy (Bowden and Holmes 2019). Otherwise, there can be a directional horizontal pleiotropy issue, or the InSIDE assumption is violated. The second method is the MR Pleiotropy Residual Sum and Outlier test (MR-PRESSO). The MR-PRESSO is developed by Verbanck et al. (2018) and it has a “global test” for assessing the overall horizontal pleiotropy. This global test uses a “leave-one-out” approach to evaluate whether a specific SNP instrument is driving the difference in computed residual sum of squares (RSS) against simulated expectations (Ong and MacGregor 2019). Finally, we made funnel plots based on the used estimator. A symmetrical plot can be evidence of no horizontal pleiotropy or balanced horizontal pleiotropy (Bowden and Holmes 2019; Richmond and Smith 2022).

According to a strategy by Jiang et al. (2022), we estimated the causal effects separately with two estimators according to the detected directional horizontal pleiotropy. The inverse-variance weighted (IVW), the most efficient estimator (Burgess et al. 2019) that can return unbiased estimates under no directional horizontal pleiotropy (Bowden et al. 2016b), was employed if no significant directional pleiotropy was detected. The IVW uses the idea of meta-analysis to offer an estimate of the causal effect by combining the Wald ratio of corresponding SNPs (Bowden et al. 2016a; Yew et al. 2020).

If a directional pleiotropy was detected, the MR-Egger regression was employed as an alternative, because it can give causal effect estimates that are robust to some directional pleiotropies (uncorrelated pleiotropy) (Morrison et al. 2020; Sanderson et al. 2019). The MR-Egger was motivated by a method in the meta-analysis literature for the assessment of small-study bias, which performs a weighted linear regression of the gene-outcome coefficients on the gene-exposure coefficients (Bowden et al. 2016a), and the regression slope coefficient of the regression provides an estimate of the causal effect (Bowden et al. 2015). Notably, we only employed the random-effect IVW as it may balance the pooled heterogeneity and offer more robust results (Chen et al. 2022b).

### 2.5. Sensitivity Analysis

We performed the MR Steiger directionality test to examine whether our assumed causal directions between the exposure and outcomes were valid (Xiao et al. 2022a; Xiao et al. 2022b). Furthermore, we conducted a sensitivity analysis for the MR-Steiger test (Two sample MR-Steiger). This sensitivity analysis can adjust measurement error and offer a sensitivity ratio (R) to indicate the reliability of the test result. A sensitivity ratio (R) over 1 can be a sign in favorable to the inferred direction of causality (Hemani et al. 2017). For associations detected with potential reverse causality, we performed the Steiger filtering method to remove potentially problematic IVs and examine if the detected direction was changed. After that, we performed Cochran’s Q test for assessing heterogeneity (Chen et al. 2022b). We also performed the MR-PRESSO to adjust the estimates after detecting and removing outliers with potential pleiotropy. Finally, we performed a leave-one-out (LOO) analysis to assess whether the estimates were significantly altered by a single IV. 

All statistical analyses were implemented using the TwoSampleMR (v 0.5.6) package in the R program (v 4.2.1). Given the multiple testing, we used an adjusted threshold (0.050/exposure/12 outcomes = 0.004) of statistical significance by the Bonferroni correction. A *p*-value smaller than 0.004 was considered significant, and between 0.004 and 0.050 was considered suggestive or potential evidence for a causal association (Cornish et al. 2020; Liu et al. 2019).

## 3. Results

### 3.1. Quality Control and Pleiotropy Diagnosis

After the clumping process and manual screening, we found no weak instrument (F < 10) throughout the quality control process. As shown in Table 2, the MR-Egger intercept test and MR-PRESSO did not reveal significant horizontal pleiotropy (global or directional). The funnel plots were also generally symmetrical (Figure S1), indicating a low risk of directional horizontal pleiotropy. Therefore, our outcomes were all processed by the IVW estimator.

### 3.2. MR Estimation

**Figure 2:**
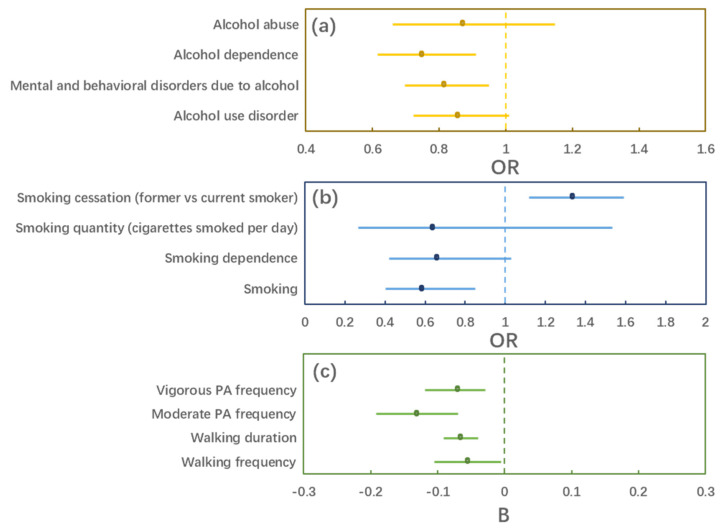
The forest plots of the causal effects. (**a**–**c**) are the effects of intelligence on alcohol, smoking, and physical activity behaviors, respectively; B, regression coefficients; OR, odds ratios.

We found significant effects of intelligence on alcohol dependence (OR = 0.749, *p* = 0.003) and smoking cessation (OR = 1.334, *p* = 0.001), and also potential effects of intelligence on mental and behavioral disorders due to alcohol (OR = 0.814, *p* = 0.009) and smoking (OR = 0.585, *p* = 0.005) (Table 3 and Figure 2). Meanwhile, we found significant effects of intelligence on walking duration (B = −0.066, *p* < 0.001), moderate PA frequency (B = −0.131, *p* < 0.001), and vigorous PA frequency (B = −0.070, *p* = 0.001). We also found suggestive evidence for a potential effect of intelligence on walking frequency (B = −0.055, *p* = 0.031). However, these associations between intelligence and PA outcomes were all in a negative direction.

### 3.3. Sensitivity Analysis

The MR-Steiger directionality test indicated that our assumed causal directions between intelligence and behavioral outcomes were accurate (Steiger *p* < 0.001). However, the sensitivity analysis adjusted for measurement error revealed that some of our hypothesized causal relationships were possibly invalid, and these results were largely supported by the sensitivity ratio (R > 1) (Table S1), indicating potential bias due to reverse causality. The observed distinctive effects of intelligence on alcohol dependence, mental and behavioral disorders due to alcohol, and smoking were affected by this issue. Unfortunately, the detected reverse causal relationships were not changed after the Steiger filtering process (no ineligible IV was found).

The Cochran’s Q test did not reveal substantial heterogeneity (Table S2). Since no outlier was detected by the MR-PRESSO, the adjustment was not carried out. The LOO analysis did not show any IV that could alter the overall estimates (Figure S2). 

## 4. Discussion

Owing to the advantage of the Mendelian randomization approach in eliminating confounding effects, emerging studies were able to investigate the causal effects of intelligence on many health outcomes, such as COVID-19 infection (Li et al. 2021), mental health issues (Ohi et al. 2021), and neurodegenerative diseases (Anderson et al. 2020). However, health-related behavior is a topic rarely investigated. In response, we carried out this study and found both positive and negative associations between genetically proxied intelligence and alcohol-, smoking-, and PA-related behaviors, which may offer extra evidence for understanding the complex health impacts of human intelligence.

### 4.1. Alcohol Behaviors

We found evidence to support the protective effects of genetically proxied intelligence against alcohol dependence and mental and behavioral disorders due to alcohol. Generally, our findings are somewhat inconsistent with some observational studies. A British Cohort Study observed that higher childhood intelligence (mental ability) was related to alcohol problems and higher alcohol intake in adult life (Batty et al. 2008). Similarly, another study suggested that higher childhood cognitive ability might place both men and women at higher risk for potential alcohol abuse (Hatch et al. 2007). Some scholars have assumed that more intelligent individuals may tend to acquire and espouse evolutionarily novel values (e.g., consuming alcohol, tobacco, and drugs) than less intelligent individuals (Kanazawa and Hellberg 2010). Our finding, in contrast, is partially in agreement with another MR study, where intelligence reduced alcohol consumption (Davies et al. 2019). These findings may collectively support another theory that high intelligence enhances the care of one’s health and therefore helps prevent unhealthy lifestyles (Deary et al. 2004). 

### 4.2. Smoking Behaviors

We found that genetically proxied intelligence potentially reduced the likelihood of smoking and increased that of smoking cessation, indicating a beneficial role of intelligence. This finding may partially explain the phenomenon observed in a large population-based cohort of male adolescents, that IQ scores were lower in male adolescents who smoke compared to non-smokers and in brothers who smoke compared to their non-smoking brothers (Weiser et al. 2010). However, there is also evidence to the contrary. A cross-country study found that societies with high average IQ tended to smoke more tobacco cigarettes per capita than those with lower IQ scores (Suhaimi et al. 2019). This difference may result from different samples, as we used individual-level data. In a previous MR study, intelligence was found to increase the likelihood of smoking (Davies et al. 2019). Despite being affected by the risk of reverse causality, our results are not supporting this finding. Altogether, our findings may challenge the relevant MR study and underline the necessity of smoke prevention in populations with relatively lower intelligence levels. 

### 4.3. Physical Activity Behaviors

We found negative effects of genetically proxied intelligence on all PA variables. These findings are inconsistent with some previous studies. Batty et al. (2007) reported that higher mental ability was positively associated with exercise habits, particularly for intense activity. Likewise, a cohort study also found higher cognitive scores were associated with higher levels of PA (Anstey et al. 2009). Nevertheless, our findings are in line with the MR study by Davies et al. (2019), where intelligence reduced the frequency of moderate and vigorous PA. This counterintuitive phenomenon, however, was not further discussed by the authors. We cannot explain this either with the current study design. A possible explanation is that individuals with higher intelligence may prefer activities more rely on intelligence so that they can have advantages, such as academic work (Kornilova et al. 2009) and Esport (Toth et al. 2020). According to the isotemporal substitution theory (Grgic et al. 2018), such activities may occupy time for others, thereby reducing the frequency of PA engagement. Furthermore, since all the PA variables were about the overall activity instead of just leisure PA, these negative associations may also be explained by occupational differences. For example, individuals with higher intelligence may tend to have jobs associated with more sedentary time (e.g., office work) while those of lower intelligence tend to select physical work. However, this is merely a hypothesis that still needs research.

### 4.4. Limitations

As we mentioned in the methods, the validation of the second and third MR assumptions is nearly impossible. This can be viewed as an inherent limitation of the MR approach. We could only use some methods to partially cope with this issue and assess the potential distortion of the results. Even so, these could not guarantee infallible analysis. For example, numerous studies have used the MR-Egger intercept test to assess horizontal pleiotropy, but the interpretation of the test may need the InSIDE assumption that can be hardly tested (Nowak and Ärnlöv 2018). Although we have adopted extra methods for assessing horizontal pleiotropy, such as the funnel plots and MR-PRESSO global test, these may not entirely eliminate the risk of pleiotropy. Likewise, the MR-Steiger test is usually used for inferring causality, but it has many limitations. For instance, it can only infer causal direction after assuming the existence of one of the two causal directions (Xue and Pan 2020), and it is also affected by pleiotropy (Lutz et al. 2021) and unmeasured confounding (Lutz et al. 2022). Therefore, our findings deserve re-examinations with extra methods.

It should be noted that owing to the potential risk of reverse causality, the estimation regarding alcohol dependence, mental and behavioral disorders due to alcohol, and smoking could have been biased. Although there is a notion that reverse causality is not a problem of MR, recent evidence implies that in some cases the estimation can be invalid (Burgess et al. 2021; Richmond and Smith 2022). For example, when the genetic variant-outcome association is not mediated by exposure, but by an upstream variable of exposure that links to the genetic variant (Burgess et al. 2021). With our study design, we were not able to investigate the reason for the reverse causality. Although the Steiger test we used is not a “gold standard” for implying causality, we caution that these associations must be taken with caution given the potential risk of bias.

We only used individuals of European ancestry, so the findings may not be generalized to other populations. Moreover, although we tried to control population stratification bias, the geographical clustering of genetic variants may introduce spurious genetic associations between our exposure and outcome (Koellinger and de Vlaming 2019). Specifically, mating behavior (e.g., assortative mating) can be affected by space. Therefore, people who live in geographical proximity to each other may share similar genes, which makes genes correlate to cultural, economic, social, political, and other environmental factors (Koellinger and de Vlaming 2019). Meanwhile, there is also a possibility that gene-driven intelligence may affect personal occupation and therefore the chance of interaction with similar peers who share certain behavioral norms (e.g., drinking, smoking, and PA). This can be viewed as an environmental selection that may influence the gene-environment association. These issues cannot be tested with our current data, so we call for future research to control the potential environmental confounding.

The dataset concerning intelligence is a pooled result of different tests. This may lead to concerns about its potential heterogeneity and effectiveness in reflecting intelligence. Thereby, re-examinations with different intelligence datasets are necessary. For example, using a GWAS dataset where intelligence is measured by a specific test can be helpful.

Finally, sample overlapping is a common issue in MR that may introduce bias to the results (Burgess et al. 2016). We checked the involved cohorts relevant to our exposure and outcome and found that intelligence and PA datasets contain some participants from the UK biobank. Theoretically, the bias due to sample overlapping can be minimized by using powerful tools (for example, the F statistic should be much greater than 10) (Pierce and Burgess 2013). Fortunately, our tools were strong enough. According to Anderson et al. (2020), any bias should be minimal under the genome-wide significance threshold we have employed (*p* < 5 × 10^−8^). Therefore, these findings could, to a certain extent, offer valuable information on this topic.

## 5. Conclusions

The presented study aimed to examine the causal effects of human intelligence on alcohol-, smoking-, and PA-related behaviors using the mendelian randomization strategy. We found evidence indicating the beneficial effects of intelligence on some alcohol and smoking behaviors. Unexpectedly, intelligence appeared to reduce the levels of PA outcomes. These findings, however, are in line with previous MR studies. Generally, our study offers extra evidence to support previous observational studies and reinforce some existing notions. Given our limitations, some findings should be understood with caution and future re-examinations are warranted.

## Figures and Tables

**Table 1:** Data sources.

GWAS ID	N	Item
ebi-a-GCST006250	269,867	Intelligence
finn-b-AUD	218,792	Alcohol use disorder (based on the International Classification of Diseases)
finn-b-F5_ALCOHOL	216,759	Mental and behavioral disorders due to alcohol
finn-b-F5_ALCOHOL_DEPENDENCE	211,535	Alcohol dependence
finn-b-KRA_PSY_ALCOH	218,792	Alcohol abuse
finn-b-SMOKING	138,088	Smoking
finn-b-SMOKING_DEPEND	218,433	Smoking dependence
ieu-a-961	68,028	Smoking quantity (cigarettes smoked per day)
ieu-a-963	70,675	Smoking cessation (former vs. current smoker)
ukb-b-4886	454,783	Walking frequency
ukb-b-16998	395,831	Walking duration
ukb-b-4710	440,266	Moderate PA frequency
ukb-b-151	440,512	Vigorous PA frequency

Note: PA, physical activity.

**Table 2:** Directional pleiotropy diagnosis.

Outcome	MR-PRESSO (Global Test)	MR-Egger	
	*p*	Intercept	*p*
Alcohol use disorder	0.682	−0.005	0.557
Mental and behavioral disorders due to alcohol	0.854	−0.0008	0.916
Alcohol dependence	0.845	−0.012	0.236
Alcohol abuse	0.774	0.010	0.462
Smoking	0.976	−0.006	0.768
Smoking dependence	0.907	−0.004	0.862
Smoking quantity	0.965	−0.007	0.876
Smoking cessation	0.842	0.001	0.894
Walking frequency	0.089	−0.002	0.399
Walking duration	0.910	−0.0008	0.461
Moderate PA frequency	0.068	−0.002	0.509
Vigorous PA frequency	0.403	−0.002	0.294

**Table 3:** Causal effects of intelligence on behavioral outcomes.

Outcome	OR/B	95% CI	*p*
Alcohol use disorder	0.855	(0.724, 1.011)	0.066
Mental and behavioral disorders due to alcohol	0.814	(0.697, 0.950)	0.009
Alcohol dependence	0.749	(0.615, 0.911)	0.003
Alcohol abuse	0.870	(0.660, 1.147)	0.323
Smoking	0.585	(0.402, 0.851)	0.005
Smoking dependence	0.658	(0.421, 1.029)	0.066
Smoking quantity (cigarettes smoked per day)	0.638	(0.265, 1.535)	0.316
Smoking cessation (former vs. current smoker)	1.334	(1.118, 1.593)	0.001
Walking frequency	−0.055	(−0.105, −0.005)	0.031
Walking duration	−0.066	(−0.091, −0.040)	<0.001
Moderate PA frequency	−0.131	(−0.192, −0.070)	<0.001
Vigorous PA frequency	−0.070	(−0.119, −0.029)	0.001

Note: Coefficients are standardized linear regression coefficients (B) or odds ratios (OR) with 95% confidence intervals (CI).

## Data Availability

Not applicable.

## Supplementary Materials

The following supporting information can be downloaded at: https://www.mdpi.com/article/10.3390/jintelligence11020029/s1, Figure S1: Funnel plots. Note: number 1 to 12 respectively represents alcohol use disorder, mental and behavioral disorders due to alcohol, alcohol dependence, alcohol abuse, smoking, smoking dependence, smoking quantity, smoking cessation, walking frequency, walking duration, moderate pa frequency, and vigorous pa frequency. Green lines indicate the IVW estimation; Figure S2: LOO plots. Note: number 1 to 12 respectively represents alcohol use disorder, mental and behavioral disorders due to alcohol, alcohol dependence, alcohol abuse, smoking, smoking dependence, smoking quantity, smoking cessation, walking frequency, walking duration, moderate pa frequency, and vigorous pa frequency; Table S1: The Steiger test and sensitivity analysis; Table S2: The Cochran’s Q test.
