# Supplementary material for: Can Intelligence Affect Alcohol-, Smoking-, and Physical Activity-Related Behaviors? A Mendelian Randomization Study
Source: J Intell. 2023 Jan 31;11(2):29. doi: 10.3390/jintelligence11020029 (PMC9968073; doi:10.3390/jintelligence11020029)
Supplement: Supplementary file 1 [file jintelligence-11-00029-s001.zip › jintelligence-2071372-supplementary.pdf]

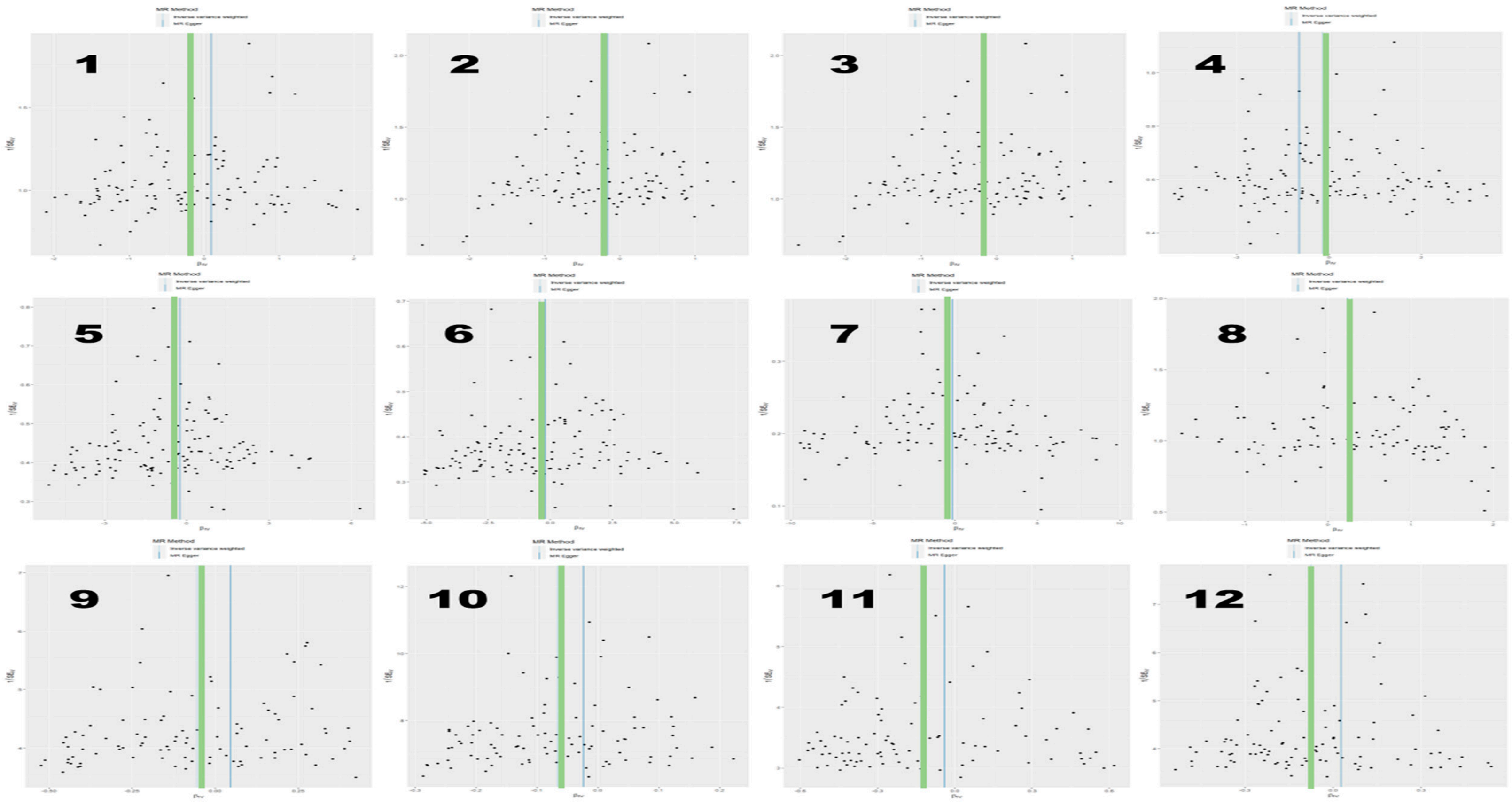

Figure S1. Funnel plots

Note: number 1 to 12 respectively represents alcohol use disorder, mental and behavioral disorders due to alcohol, alcohol dependence, alcohol abuse, smoking, smoking dependence, smoking quantity, smoking cessation, walking frequency, walking duration, moderate pa frequency, and vigorous pa frequency. Green lines indicate the IVW estimation.

Table S1. The Steiger test and sensitivity analysis.

Outcome	Correct-causality	Sensitivity Ratio	Reliability of the test
	Adjusted for measure error	R	
Alcohol use disorder	T	1.221	Yes
Mental and behavioural disorders due to alcohol	F	1.154	Yes
Alcohol dependence	F	1.493	Yes
Alcohol abuse	F	3.783	Yes
Smoking	F	15.407	Yes
Smoking dependence	F	33.912	Yes
Smoking quantity (Cigarettes smoked per day)	F	-6683.658	No
Smoking cessation (Former vs current smoker)	T	1.221	Yes
Walking frequency	T	12.678	Yes
Walking duration	T	33.664	Yes
Moderate PA frequency	T	8.895	Yes
Vigorous PA frequency	T	15.189	Yes

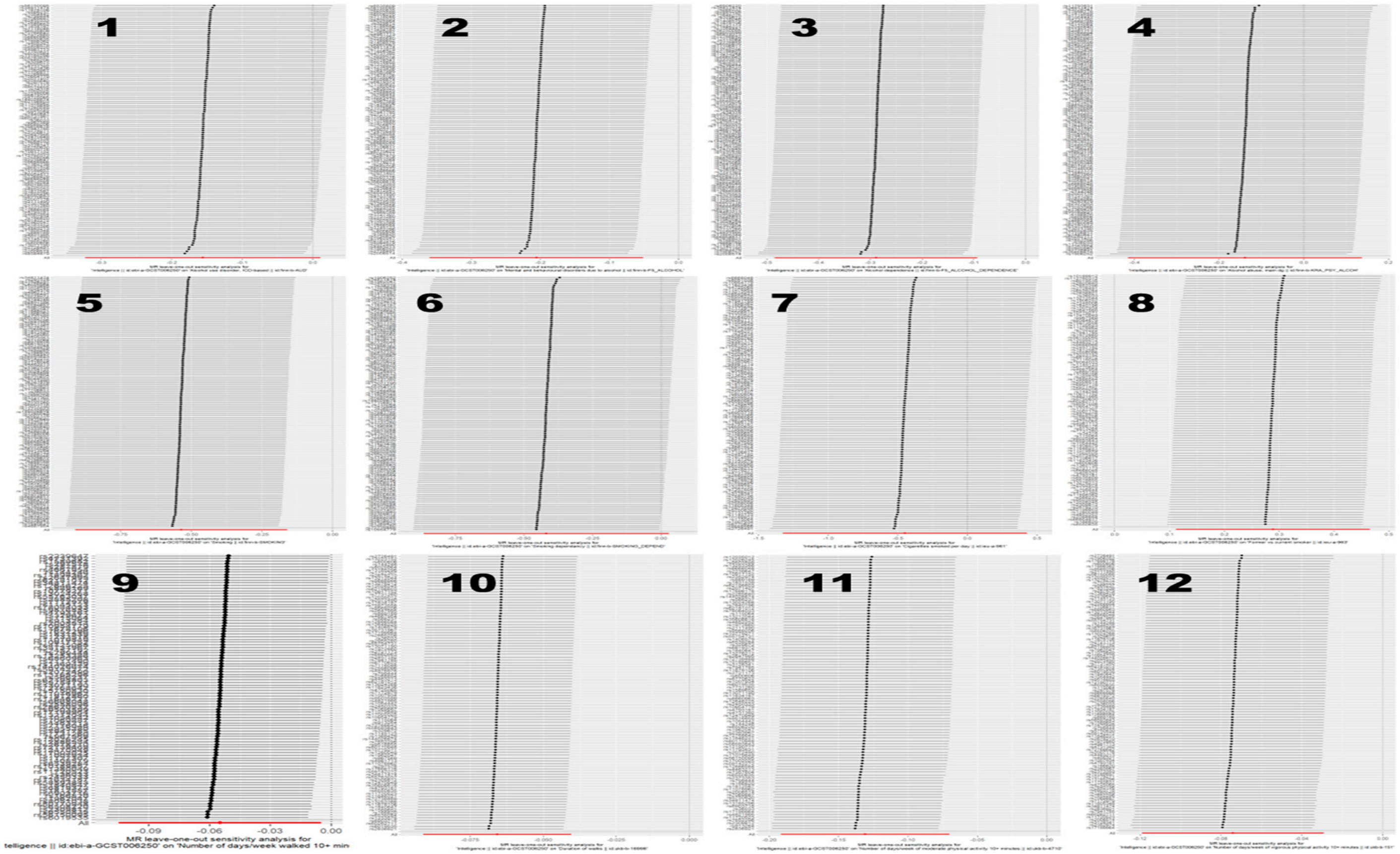

Figure S2. LOO plots.

Note: number 1 to 12 respectively represents alcohol use disorder, mental and behavioral disorders due to alcohol, alcohol dependence, alcohol abuse, smoking, smoking dependence, smoking quantity, smoking cessation, walking frequency, walking duration, moderate pa frequency, and vigorous pa frequency.

Table S2. The Cochran's Q test

Outcome	Cochran's Q	*p*
Alcohol use disorder	111.183	0.659
Mental and behavioural disorders due to alcohol	98.211	0.838
Alcohol dependence	111.991	0.857
Alcohol abuse	14.653	0.776
Smoking	104.498	0.972
Smoking dependence	109.002	0.887
Smoking quantity (Cigarettes smoked per day)	82.839	0.960
Smoking cessation (Former vs current smoker)	87.153	0.817
Walking frequency	114.743	0.093
Walking duration	81.178	0.904
Moderate PA frequency	117.369	0.060
Vigorous PA frequency	102.998	0.389
